# Ectopic TSH-secreting pituitary tumor: a case report and review of prior cases

**DOI:** 10.1186/1471-2407-14-544

**Published:** 2014-07-28

**Authors:** Mingqiang Song, Haijing Wang, Li Song, Haiye Tian, Quanxu Ge, Jun Li, Yan Zhu, Jizhou Li, Runzhen Zhao, Hong-Long Ji

**Affiliations:** Department of Endocrinology, Weihai Municipal Hospital, 70 Heping Road, Weihai, Shandong 264200 China; Department of Neurosurgery, Hebi First People’s Hospital, Hebi, Henan 458030 China; Department of Pathology, First Affiliated Hospital of Nanjing Medical University, Nanjing, Jiangsu 210029 China; Department of Cellular and Molecular Biology, University of Texas Health Science Center at Tyler, Tyler, TX 75708 USA

**Keywords:** Ectopic TSH-secreting pituitary adenoma, Resistance to thyroid hormone (RTH), TRH stimulating test, Octreotide inhibition test, Hyperthyroidism

## Abstract

**Background:**

Ectopic TSH-secreting pituitary adenoma (TSH-oma) is a very unusual disorder. To date, there are only four cases reported. It is difficult to distinguish ectopic cases from both regular TSH-omas and resistance to thyroid hormone (RTH).

**Case presentation:**

A newly identified case of ectopic TSH-oma arising from the nasal pharynx was described, and reports of four prior cases were reviewed. The patient was a 41-year-old male who developed what appeared to be typical hyperthyroidism and atrial fibrillation in 2009. Thyroid function tests showed elevated basal levels of free T_3_ (FT_3_, 24.08 pmol/L), free T_4_ (FT_4_, 75.73 pmol/L), and serum TSH (7.26 μIU/ml). Both TSH-oma and resistance to thyroid hormone syndrome were considered. TRH stimulating test was negative, whereas octreotide inhibition test showed a reduction in TSH by 30.8%. Furthermore, a large space-occupying lesion located at the nasopharynx was found by computed tomography and magnetic resonance imaging (MRI). A normal pituitary was visualized. Ectopic TSH-oma was preliminarily established. Using an endoscopic endonasal approach, the tumor was resected. Histological features and immunophenotypes were consistent with those of TSH-secreting tumor. The levels of both free thyroxine and TSH returned to normal ranges the day after surgery and remained within normal range for 48 months.

**Conclusions:**

Although exceedingly rare, ectopic TSH-oma should be considered for patients with inappropriate secretion of TSH with hyperthyroidism and pituitary tumor undetectable by computed tomography and MRI. To our knowledge, this is the first case followed up more than 4 years. The characteristics and successful interventions summarized in this report provide a guideline for clinicians.

## Background

TSH-secreting pituitary adenomas (TSH-omas) are an unusual disorder, accounting for ~2% of all pituitary tumors [1]. Ectopic TSH-oma is extremely rare. Since the first description of the disease by Cooper and colleagues in 1996, only four cases have been reported to date [2–5]. Here a newly identified case is reported, and the clinical and laboratory features of previous published cases are reviewed.

## Case presentation

**Figure 1:**
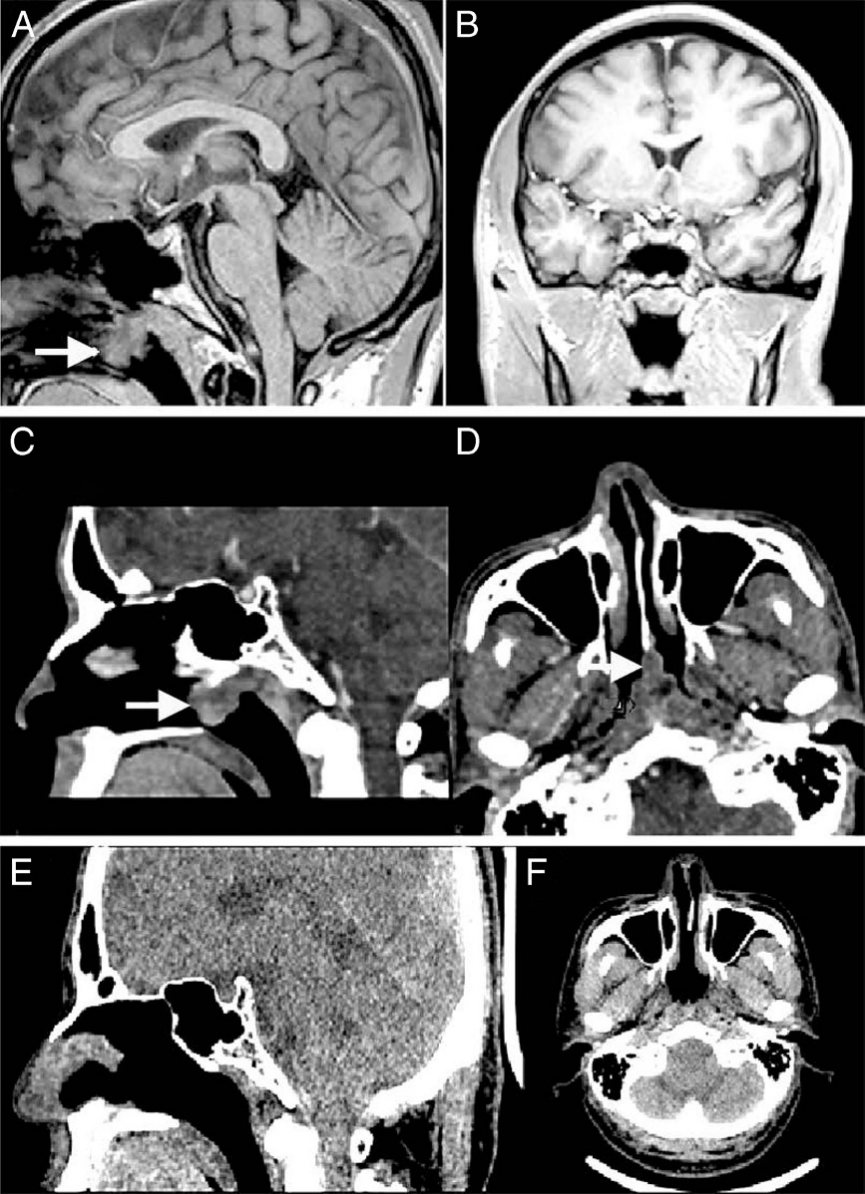
**MRI and CT images. A** &**B**, MRI of the pituitary showing a normal pituitary gland and an ectopic pituitary tumor in the nasopharynx (white arrow). **C** &**D**, CT scan showing a 1.9 × 1.7 cm mass in the nasopharyngeal cavity (white arrows). **E** &**F**, CT scan 48 months post surgery.

A 41-year-old male suffering from palpitations, dyspnea, weight loss, and fatigue for one year was referred to Weihai Municipal Hospital in June 2009. He also had atrial fibrillation. Thyroid functional tests showed increased FT_3_ (24.08, normal 2.8-7.1 pmol/L), FT_4_ (75.73, normal 12-22 pmol/L), and TSH (7.26, normal 0.27-4.2 μIU/ml). He was diagnosed with hyperthyroidism and given propylthiouracil (300 mg daily) together with either propranolol or propafenone. The patient's electrocardiogram displayed sinus rhythm. The levels of FT_3_ and FT_4_ (FT_3_ 11.54 pmol/L, FT_4_ 27.09 pmol/L) but not TSH (14.08 μIU/ml) were reduced after six months of treatment. However, the concentration of free thyroid hormones were still not normal. In sharp contrast, the TSH level was further elevated after intensive treatment. Pituitary MRI examination was therefore performed to rule out TSH-oma. The MRI image indicated a normal pituitary gland (Figure 1A and B). Thus, resistance to thyroid hormone syndrome was diagnosed, and triiodothyroacetic acid was prescribed. The plasma levels of FT_3_, FT_4_, and TSH transiently decreased and then rebounded.

**Figure 2:**
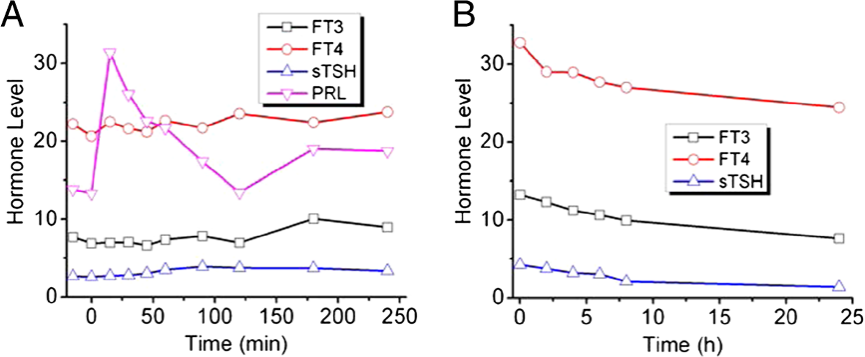
**TRH stimulating and octreotide inhibition tests.** The unit for FT_3_ and FT_4_ is pmol/L, for PRL is ng/ml, and for sTSH is μIU/ml. **A**, TRH stimulating test. **B**, Octreotide inhibition test.

Over the course of the disease, the patient lost 6 kg of body weight. He had no symptoms of headache, nausea, dizziness, subnormal vision, impaired visual field, and obvious nasal obstruction. His physical examination was normal (T 36.7°C, P 80/min, R 17/min, BP120/80 mmHg, Ht 173 cm, Wt 65 Kg). He had a symmetrical figure, normal hair distribution, sweaty skin, normal superficial lymph nodes, and normal degree of convexity of eyeballs. Palpation revealed swelling of the thyroid gland, no nodules, medium texture, and no haphalgesia. Vascular murmur was not heard on auscultation. The patient had uneven cardiac sounds and arrhythmia with a heart rate of 100/min. No abdominal abnormalities were found. The proximal muscles did not show signs of atrophy. Mild tremor was observed when he raised his hands. The patellar tendon reflex was normal, and the pathological reflex was not observed. Lab and imaging results showed normal liver and kidney. TG-Ab <30%, TM-Ab <15%, GH 0.7 (normal <5.0 ng/ml), FSH 16.8 (normal 1.5-12 mIU/ml), LH 13.72 (normal 1.7-8.6 mIU/ml), PRL 14.9 (normal 4.1-18.4 ng/ml), and T 13.20 (normal 2.8-8.0 ng/ml). The blood electrolytes were within normal ranges: Ca 2.27 mmol/L, P 1.23 mmol/L, and K 3.87 mmol/L. Tumor biomarkers were analyzed, including carbohydrate antigen-199 12.26 (normal <37 U/ml), carcinoembryonic antigen 1.90 (normal <10 ng/ml), and neuron specific enolase (NSE) 22.31 (normal <16.3 ng/ml). Given a normal pituitary gland as pictured by magnetic resonance imaging (MRI) with gadolinium contrast, abnormal TSH level, and a large space-occupying lesion within the nasal cavity and the nasopharynx, with a maximum cross-section area of 1.9 × 1.7 cm (Figure 1C & D), as detected by CT scan, an ectopic TSH-secreting pituitary tumor was suspected. Emission computed tomography (ECT) demonstrated strong technetium-uptake by the thyroid. However, as shown in Figure 2A, the stimulating test was negative for TSH level was not up-regulated by TRH (Shanghai Lizhudongfeng Biologic Technology Inc., China). The amount of TSH was increased <2 μIU/ml. In comparison, the octreotide inhibition test was positive; a decrease of up to 30.8% in TSH level was observed (Figure 2B). Taken together, a diagnosis of ectopic TSH-secreting tumor in the nasopharynx was tentatively established. The strategy to combine resection and quick pathological examination was proposed to release patient’s financial burden and surgical stress. Nevertheless, his surgery was postponed due to treatment of the atrial fibrillation.

**Figure 3:**
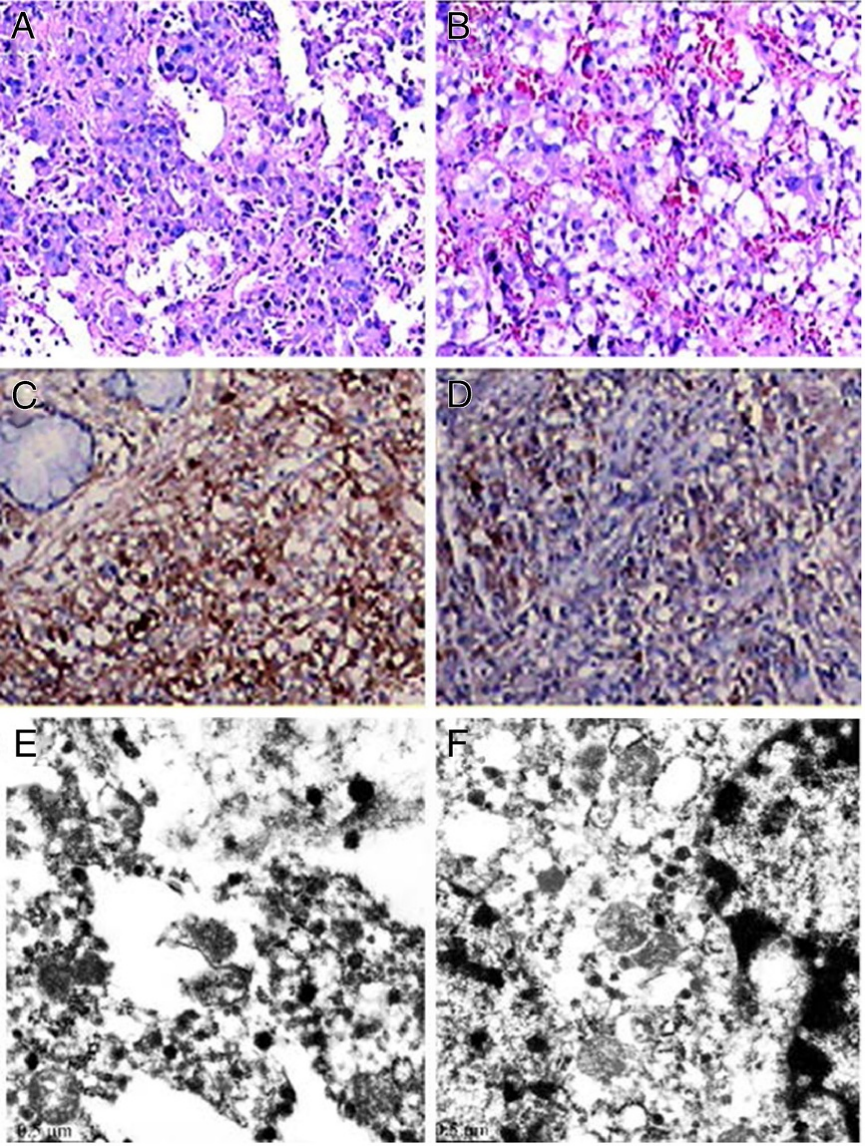
**Histological examination of resected tumor tissue. A** &**B**, Histological examination of resected tumor tissue (×200). **A**, Irregular cells showing tumor tissue invasive growth involving the submucosa. **B**, Cytoplasm is filled with fine granules in tumor cells. **C** &**D**, Immunohistochemical detection of TSH and GH (×200). **C**, Most tumor cells express TSH (brown). **D**, Expression of GH in tumorous cells. **E** &**F**, Electron microscopy examination of tumor tissue. Numerous round electron dense granules about 100-200 nm in size are seen in the cytosol. Bar = 0.5 μm.

The patient underwent endoscopic endonasal surgery, through which the mass was removed in November 2009. Pathological examination confirmed invasive ectopic pituitary tumor extending to the bone parenchyma. Microscopic examination showed that the unenveloped tumor tissue lay beneath the nasal mucosa (pseudostratified ciliated columnar epithelium) (Figure 3A & B). The tumor cells invading the mucosa and fibrous tissue led to a tumor tissue type that was diffusive, solid lesion, and sinusoidal. The irregular tumor cells were mostly round or polygonal, while others were spindle shaped, filled with rich cytoplasm bearing fine particles. Some cells were found to have a round to ovoid nucleus with transparent cytoplasm (Figure 3A & B). Immunohistochemical assays detected expression of TSH (brown) and GH (brown) (Figure 3C & D). No other pituitary hormones, including ACTH, LH, FSH, and PRL, or thyroglobulin and thyroid transcription factor1 were detectable. In contrast, pan-cytokeratin, chromogranin, synaptophysin, and neuron specific enolase were detected. Electron microscopy examination found that the tumor was composed of pleomorphic cells (Figure 3E and F). Round, unenveloped electron-dense granules were dispersed throughout the cytoplasm. Some granules formed clusters. The diameter of granules was 0.1-0.2 μm.

Upon resection of the tumor, the levels of plasma TSH, FT_3_, and FT_4_ returned to normal ranges, as analyzed 24 hours post surgery (Table 1). The patient gained 3 kg of body weight in two months. In addition, the symptoms of sweating, palpitation, and fatigue disappeared. Atrial fibrillation was treated with metoprolol and warfarin. We re-examined plasma TSH, FT_3_ and FT_4_ levels 48 months post surgery and found them still normal (Table 1). Meanwhile, CT scan did not indicate recurrence of the tumor (Figure 1E & F).

**Table 1:** **Plasma thyroid hormone and TSH levels**

Date	T _3_	T _4_	FT _3_	FT _4_	TSH	Remarks
	(0.89–2.44)	(62.67–150.84)	(2.80–7.10)	(12.0–22.0)	(0.27–4.20)	
2009-02-14			24.80	75.73	7.26	PTU 300 mg/d
2009-06-08			11.54	27.09	14.08	PTU 300 mg/d
2009-07-12	4.23	191.23	14.30	29.86	5.72	PTU 300 mg/d
2009-11-12			4.85	13.54	1.86	24 h post surgery
2013-11-17			5.28	19.26	0.65	48 m post surgery

The normal range for each assay is included in brackets.

The unit for T_3_ and T_4_ is nmol/L, for FT_3_ and FT_4_ is pmol/L, and for TSH is μIU/ml.

### Discussion

Ectopic TSH-omas are extremely rare. To date, only four cases have been reported. In all five cases of ectopic TSH-oma, including our patient, the tumor was located in the nasopharynx. Their clinical and laboratory features are summarized in Table 2.

**Table 2:** **Comparison of five ectopic TSH-omas**

Case number	First	Second	Third	Fourth	Fifth
Reference	2	3	4	5	This report
Gender	F	M	F	F	M
Age of onset (y)	45	34	-	34	40
Age of diagnosis	66	52	50	49	41
Location of tumor	nasopharynx				
Follow up	no recurrence at 2 months	recurrence at 10 months	no recurrence at 4 months	no recurrence at 3 months	no recurrence at 48 months
IHC	TSH(+)	TSH(+)	TSH(+)	TSH(+)	TSH(+)
	GH(+)	GH(-)	GH(+)	GH(+)	GH(+)
	PRL(+)	PRL(-)	PRL(-)	PRL(+)	PRL(-)
	FSH(+)	FSH(-)	FSH(+)	FSH(-)	FSH(-)
	ACTH(+)	ACTH(-)	ACTH(-)	ACTH(-)	ACTH(-)
	LH(+)	LH(-)	LH(+)	LH(-)	LH(-)
Ultrastructure	N/A	N/A	N/A	consisted of monomorphous cells with secretory granules of small thyrotroph-like cells	consisted of polymorphous cells with secretory granules
Octreotide inhibition test	N/A	N/A	N/A	yes	yes
TRH stimulating test	N/A	N/A	N/A	N/A	yes

This patient was diagnosed as ectopic TSH-oma in November 2009. Similar to the other cases, the patient went to see the doctor for hyperthyroidism with diffuse goiter and atrial fibrillation; ophthalmopathy, pretibial myxedema, and periodic paralysis were not presented. Additionally, the previous four cases had a common specific symptom of airway obstruction resulting from space occupying effects. Nevertheless, it was not evident in this patient, leading to overlook of existence of tumor by both the patient and physicians.

With regard to the phylogenetics of ectopic pituitary adenoma, it is broadly accepted that the tumor is derived from the embryonic residues of pituitary cells along the path of migration of Rathke's pouch. The anterior pituitary primordium appears at the fourth week of embryogenesis. The pituitary then divides into sellar and pharyngeal parts in the eighth week. The craniopharyngeal canal allows for migration of the pituitary tissue into the sphenoid sinus/bone or nasopharynx. Nasopharyngeal and sphenoid sinus or sphenoid bone ectopic pituitary tissue can be fully functional, since pharyngeal pituitary tissue begins to produce hormones around the 17-18th week of gestation (about 8 weeks later than sellar pituitary function begins) [6]. Landolt and co-workers found that 90 - 100% of adults had ectopic pituitary tissue in the sphenoid sinus/bone [7]. The pharyngeal hypophysis released all six normal pituitary hormones (ACTH, TSH, PRL, LH, FSH, and GH) [8]. It is postulated that the embryonic residues of pituitary cells produce tumor lesion, and synthesize pituitary hormones.

Of note, the first case received radioactive iodine treatment without prior measurement of the TSH level. Although hyperthyroidism was abrogated, the consequence was hypothyroidism with an increased level of TSH. This obscured the nature of the disease and complicated the diagnostic process. This was the only case that received radiation therapy for the thyroid prior to final diagnosis. Therefore, it was difficult to determine whether the tumor was a primary ectopic TSH-secreting tumor or resulted from radioiodine thyroid ablation-induced hypothyroidism. Remission of the latter could be achieved by administration of thyroid hormones. Indeed, invasive transformation of the tumor and high occurrence of invasive macroadenomas were described in patients with previous thyroid ablation by surgery or radioiodine. It resembled the occurrence of Nelson’s syndrome after adrenalectomy for Cushing’s disease.

Ectopic TSH-oma and pituitary TSH-secreting tumor in the sellar area cannot be differentiated by their biological characteristics. Both present high levels of serum FT_3_ and FT_4_, in addition to either normal or high level of TSH. The difference between these two tumor types is that the ectopic TSH-oma has a normal pituitary gland and sellar turcica. Nowadays, with high-resolution CT and MRI, large pituitary adenomas are easy to find; moreover, it is not difficult to detect micro-adenomas either [9, 10].

**Figure 4:**
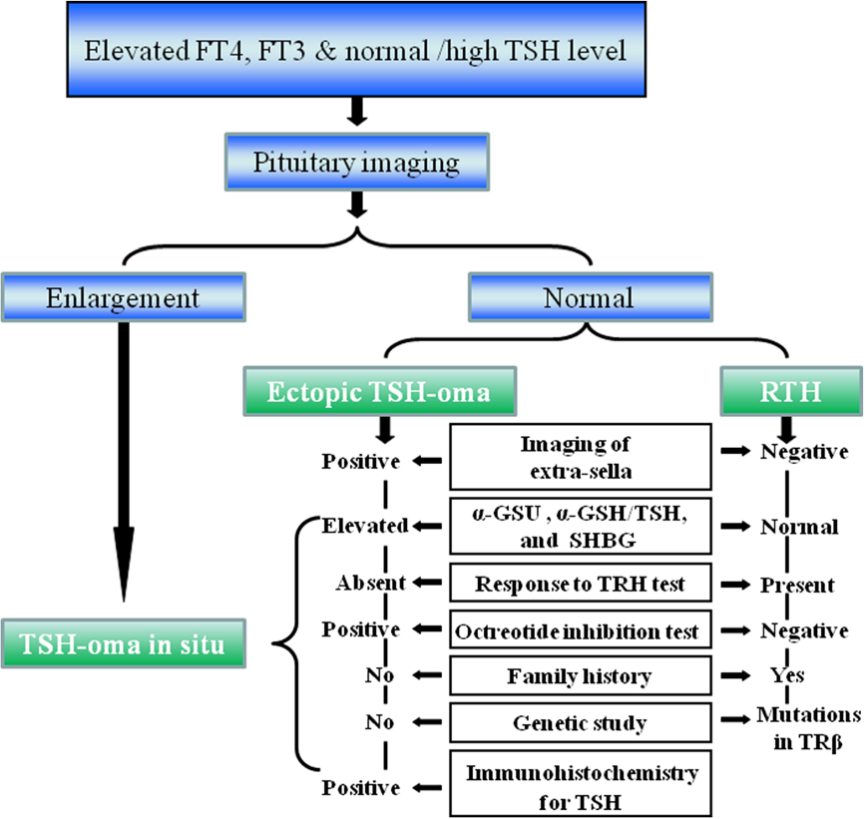
**Differential diagnosis of TSH-omas.** α-GSU, α-glycoprotein hormone subunits; SHBG, sex-hormone binding globulin; TRH, thyroptroin releasing hormone; TRβ, thyroid hormone receptor β.

It is not easy to distinguish TSH-oma from resistance to thyroid hormones (RTH) (Figure 4). RTH is rare, more than 90% of RTH are hereditary, displaying autosomal dominant inheritance, which are linked to mutations of thyroid hormone receptor β gene. RTH also exhibits high FT3 and FT4 levels and inappropriate TSH secretion. In addition, there were no significant differences in the basal values of TSH and free thyroid hormones between TSH-secreting tumor and RTH [11, 12]. Hence, other diagnostic measures are required. Glycoprotein hormone subunits (α-GSU) and molar ratio of α-GSU/TSH are valuable indicators to distinguish TSH-secreting tumor from RTH. More than 80% of TSH-secreting tumors had hypersecretion of circulating free α-GSU and an elevated α-GSU/TSH molar ratio [9, 12, 13]. It was more common in macroadenomas than in micro-adenomas [9]. The pituitary adenoma causing hyperthyroidism is composed of two types of cells, one secreting α-GSU alone, and the other producing both α-GSU and thyrotropin but not in equal amounts [14]. Generally, α-GSU is secreted more than TSH. However, in this case, α-GSU was not detected. Furthermore, TSH-oma displayed an elevation in sex-hormone-binding globulin, while it was normal in RTH [12]. The final diagnosis was made by TRH stimulating and octreotide inhibition tests. While 96% of TSH-secreting tumor presented a blunted TSH response to the TRH test and 97% of RTH were excited by TRH [12]. This patient presented a blunted TSH response to the TRH test (Figure 2A). Most pituitary TSH-secreting tumor cells possess somatostatin receptors, which are sensitive to somatostatin and its analogues. FT3 and FT4 levels decreased markedly following delivery of somatostatin analogues in all TSH-omas but not RTH patients [12]. Similarly, the inhibitory effect of octreotide was seen in ectopic TSH-oma too [5]. This patient presented a significant inhibitory response to octreotide (Figure 2B), and the inhibitory effect of octreotide on ectopic TSH-oma cells was also confirmed in vitro [5]. In addition, TSH-secreting tumor cells possessed dopamine receptors. The presence of dopamine receptors in TSH-omas was the rationale for therapeutic trials with dopaminergic agonists. Several studies, however, have shown a large heterogeneity of TSH responses to dopaminergic agents [13, 15]. In fact, administration of dopamine agonists failed to persistently block TSH secretion in almost all patients and caused tumor shrinkage only in those with combined hypersecretion of TSH and PRL [16].

TSH-omas are generally benign tumors. However, transformation of TSH-oma into carcinoma with multiple metastases and loss of pituitary α-GSU has been reported [17]. TSH-secreting carcinoma could also develop from previously non-functioning pituitary adenoma [18]. All five cases of ectopic TSH-omas had characteristics of benign tumors. Although tumors of some cases invaded into adjacent tissues, none showed distant metastasis [3].

Morphological characteristics of tumor cells were inconsistent including unitary shape, irregular morphology, and multiple types of cells. Cells contained abundant granular cytoplasm and round or oval nuclei. Relatively large amounts of blood sinuses existed in the tumor. The adenoma consisted of monomorphous cells as visualized by electron microscopy. Numerous secretory granules were scattered across the cytoplasm or along the cell membrane [5]. They were similar in size and shape of electron dense with a diameter of 60-120 [5]. In comparison, the tumor cells of this case were pleomorphic, and the size of their electron dense granules was larger with a diameter of 100-200 nm, and were scattered or clustered in the cytoplasm.

Immunohistochemical examination is essential for studying the nature of the tumor cells and hormone secretion. Almost all neuroendocrine tumors have enhanced expression of chromogranin A, synaptophysin, and neuron-specific enolase [6, 19]. Therefore, these proteins have been used as biomarkers of neuroendocrine tumor. Except for the case reported by Collie, strong expression of various neuroendocrine biomarkers, including chromogranin A, synaptophysin, and neuron-specific enolase was confirmed. As for cell proliferation, the amount of Ki-67-positive cells was less than 2%, suggesting that cell proliferation of the ectopic TSH-oma was low, in agreement with what was known of TSH-oma in situ. The types of hormones secreted by ectopic TSH-oma were not identical (Table 2). Except for the second case, GH expression in the tumor tissues was detected. In addition, augmentation of the expression of TSH and GH was also described in vitro [5]. However, the serum level of GH in the fourth patient was normal, inconsistent with biochemical changes and clinical manifestation in vivo [20, 21]. The mechanism remains unclear. It may be due to lesser secretion of secondary hormone or limited release into blood.

The therapeutic approach in all five cases was adenomectomy. The primary objectives of the surgical treatment were to remove the ectopic TSH-oma, to eliminate the excessive secretion of TSH, and to restore euthyroidism. The prerequisite was to reduce the level of thyroid hormone to ease thyrotoxicosis prior to adenomectomy. The most common strategy is to take either anti-thyroid drugs (methimazole 20-30 mg/d or propylthiouracil 200-300 mg/d) or somatostatin analogs (octreotide, 100 μg, s.c., bid or tid. Sandostatin®, Novartis Pharma Schweiz AG, Switzerland) as well as propranolol (80-120 mg/d orally). Obviously, somatostatin analogs should be preferred theoretically, and this has been borne out in practice. For example, the TSH level of the fourth case returned to normal one day post octreotide treatment (100 μg, ih, q8h). Meanwhile, the levels of FT_3_ and FT_4_ declined to normal in 7 days. In contrast, it was difficult to control TSH and thyroid function with anti-thyroid drugs. In this case, PTU (300 mg/d) could not reduce free thyroxine to normal levels (Table 1). TSH and free thyroxine levels were normal within a few days [22], even within 24 hr after surgery in our case study. Apparently, it is feasible to treat TSH-omas by in situ radiotherapy. However, this intervention was not applied to all five cases of ectopic TSH-omas [2–5].

Given that somatostatin (somatotropin release-inhibiting hormone, SRIH) receptor was expressed in TSH-omas [23, 24], somatostatin analogues have been used to treat TSH-oma in situ. Somatostatin analogues are potent in reducing TSH secretion. Long-acting somatostatin analogues, including octreotide LAR, lanreotide SR, and lanreotide autogel were preferred [9, 10, 25, 26]. These medicines decreased TSH and α-GSU secretion, and restored euthyroidism. Circulating thyroid hormone levels were normalized in more than 95% of patients, and pituitary tumor mass shrinkage occurred in approximately 40% of patients [12]. Furthermore, the efficiency of the somatostatin analogue was observed in an ectopic TSH-oma patient [5].

## Conclusions

In toto, ectopic TSH-oma is extremely rare. To date, only four cases have been reported. The phylogenetic mechanism of ectopic TSH-oma should be similar to other ectopic pituitary tumors, which are probably derived from the embryonic residues of pituitary cells along the path of migration of Rathke's pouch. All five cases were found to have a mass in the nasopharyngeal region. Their clinical manifestations were almost the same as those of ordinary hyperthyroidism (high metabolic syndrome). Nevertheless, they all had high levels of TSH as well as increased serum free thyroxine. Alpha-GSU and α-GSU/TSH ratio are valuable for distinguishing TSH-oma from other diseases. Moreover, TRH stimulating and octreotide inhibition tests could differentiate ectopic TSH-oma from RTH. The primary therapy for ectopic TSH-oma is the resection of adenoma. Nonsurgical intervention through long-acting somatostatin analogues can suppress TSH secretion. Whether in situ radiation therapy could be an effective intervention remains unknown.

## Consent

Written informed consent was obtained from the patient for publication of this Case Report and any accompanying images. A copy of the written consent is available for review by the Editor of this journal.
